# Prevalence of Diabetes and Prediabetes, and Achievements in Diabetes Control in Iran; The Results of the STEPS of 2016

**DOI:** 10.34172/aim.2022.94

**Published:** 2022-09-01

**Authors:** Saeid Shahraz, Sahar Saeedi Moghaddam, Mehrdad Azmin, Niloofar Peykari, Moein Yoosefi, Farnam Mohebi, Shahab Khatibzadeh, Sahar Mohammadi Fateh, Shirin Djalalinia, Mitra Modirian, Negar Mahmoudi, Zohreh Mahmoudi, Sarina Dashti, Alireza Mahdavi Hezaveh, Bagher Larijani, Farshad Farzadfar

**Affiliations:** ^1^Institute for Clinical Research and Health Policy Studies, Tufts Medical Center, Boston, Massachusetts, USA; ^2^Non-Communicable Diseases Research Center, Endocrinology and Metabolism Population Sciences Institute, Tehran University of Medical Sciences, Tehran, Iran; ^3^Deputy for Education, Ministry of Health and Medical Education, Tehran, Iran; ^4^Haas School of Business, University of California, Berkeley, CA 94720, USA; ^5^Brandeis University, Heller School for Social Policy and Management, Waltham, Massachusetts, USA; ^6^Endocrinology and Metabolism Research Center, Endocrinology and Metabolism Clinical Sciences Institute, Tehran University of Medical Sciences, Tehran, Iran; ^7^Deputy of Research and Technology, Ministry of Health and Medical Education, Tehran, Iran; ^8^Center for Noncommunicable Disease Control and Prevention, Ministry of Health and Medical Education, Tehran, Iran

**Keywords:** Control, Prediabetes, Prevalence, Risk factors, Surveys

## Abstract

**Background:**

Using the WHO STEPwise approach to NCD risk factor surveillance (STEPS), first round of Iran’s STEPS completed in 2005. It has been repeated six times afterward. Here we report the results of 2016 round on the population characteristics and prevalence of diabetes and prediabetes, along with an assessment of the country-level performance on diabetes care in Iran.

**Methods:**

Using a proportional-to-size cluster random sampling method, the STEPS 2016 included 18947 subjects aged≥25 years who matched the criteria (non-missing information on diabetes self-report, and biomarkers). For the analyses, survey design methods with weighted samples were employed. Different definitions of diabetes (biomarker-based, self-report, anti-diabetes medication use, or a combination) and prediabetes (different cutpoints of the biomarker) were calculated and presented.

**Results:**

An estimated 5171035 persons aged≥25 years or 10.6% (95% CI: 10.0%–11.1%) had diabetes according to the serologic diagnosis of diabetes (FPG≥126 mg/dL) or the use of at least one anti-diabetes medication (1896 out of 18947). Employing the serologic diagnosis of diabetes among those who responded no to the self-reported question, 2.7% (2.5%–3.0%) of the population were not aware of their diabetes compared to 11.5% (10.9%-12.0%) who were diabetics according to the just self-reported question. Defining prediabetes as 100≤FPG<126 mg/dL or 5.7≤HbA1c<6.5%, an estimated 15244299 persons had prediabetes (5885 out of 18947). Overall, 52.1% (49.4%–54.7%) of patients with self-reported diabetes were under strict glycemic control (HbA1c<7%). Poor diabetes control (HbA1c>9%) was found in 18.4% (16.3%-20.6%) of the patients with self-reported diabetes.

**Conclusion:**

Since 2005, the prevalence of diabetes in Iran has been on a gradual increase in both genders with an increasing gap between females and males.

## Introduction

 Rapid health transition from infectious diseases toward non-communicable conditions^1^ placed diabetes among the leading causes of morbidity and mortality in Iran and its neighboring countries in 2010.^2,3^ The rapid increase in diabetes burden in Iran is putting extraordinary strains on the health care system and the population. Javanbakht et al estimated the direct cost of only type 2 diabetes mellitus to be 8.7% of the total health expenditure of Iran in 2009.^4^ According to a systematic review conducted by our colleagues in 2017, the national prevalence of diabetes consistently increased from 5.48% in 1990 to 9.06% in 2016. A fasting plasma glucose (FPG) of at least 126 mg/dL or self-report of receiving an anti-diabetes medication was the basis for the definition of diabetes in this review. The relative prevalence was consistently higher in women than men. In 1990, the relative prevalence of diabetes was 0.77% higher in women than men. This gender gap in prevalence continuously increased over time and was estimated at 1.49% in 2016.^5^

 Making accurate predictions of the disease burden is crucial for future decisions on proper diabetes care in the country. Therefore, Iran has opted to complete the World Health Organization’s STEPwise approach to risk factor surveillance (STEPS) periodically since 2005.^6^ STEPS is a periodical, nationally representative survey of risk factors for non-communicable diseases. This survey includes diabetes and prediabetes. The scope and methodology of the STEPS, completed at least once in more than 100 developing countries to date,^7^ are analogous to the US NHANES (National Health and Nutrition Examination Survey).^8,9^ Therefore, analysis of the STEPS data provides a unique opportunity to update the epidemiological features of diagnosed and undiagnosed diabetes and prediabetes using both HbA1c and FPG measures. Also, like NHANES, STEPS conveys information that enables the evaluation and reporting on the overall achievements of the system for diabetes care. In the current study, for the first time, we report the epidemiological features of high blood sugar and the country-level performance on diabetes care using STEPS 2016 data.

## Materials and Methods

**Figure 1:**
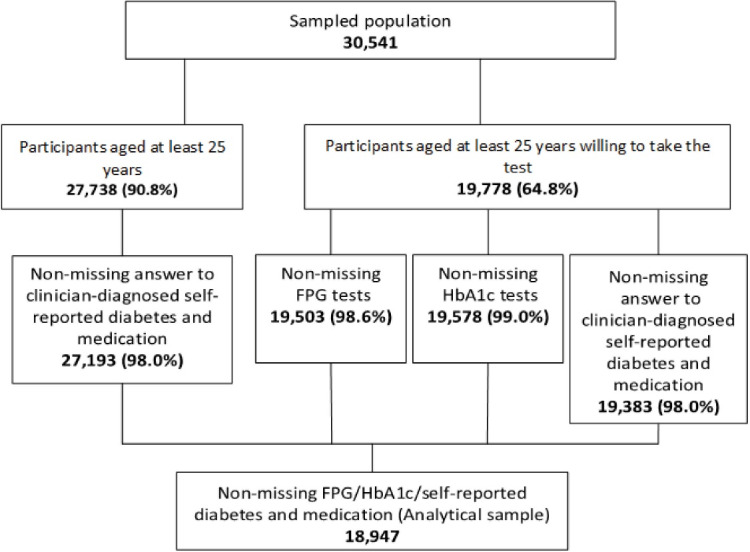
Flow Diagram that Shows the Steps of Building the Analytical File Consisting of 18 947 Individuals

 Under the auspices of the Iran Ministry of Health and Medical Education (MOHME), the Non-Communicable Diseases Research Center of Tehran University of Medical Sciences (Tehran, Iran) conducted the nationally represented STEPS study in Iran. The 2016 version of the STEPS instrument was a validated revision of the earlier questionnaires used periodically since 2005. This new version comprised five modules: behavioral, healthcare utilization, screening programs, physical examination, and laboratory assessment. The original target population included 31 050 people aged at least 18 years living in 31 provinces. The study adopted a proportional-to-size cluster random sampling method that considered the postal code of residential households as a sampling frame. Qom, a small province in central Iran with an adult population of nearly 1 million, did not participate in the sampling. Sample weights, which also accounted for non-response, were calculated for estimating the national-level prevalence. These weights were re-adjusted to eliminate the impact of dropping out of the Qom province in the sample. With a 98.4% response rate, the sample population included 30 541 participants in 389 districts for the interview survey.^6^ A large subsample of the participants of the interview module, aged at least 25 years, showed a willingness to take part in the laboratory measures. The analytical sample consisted of 18 947 observations with non-missing information on self-reported diabetes and the laboratory diagnosis of diabetes. Figure 1 conveys more details on the selection of the analytic sample and the missingness pattern of the data at each step.

 We identified patients with diabetes among those who positively answered the self-reported diabetes question. Self-reported diabetes corresponded to the question: “Has a physician or a healthcare staff ever told you that you have diabetes or high blood sugar?” Laboratory-based diagnosis of diabetes required an FPG of at least 126 mg/dL. Besides, we provided estimates of diabetes using alternative definitions that factored in the self-report of using anti-diabetes medications. While self-reported diabetes seems to be a standard way of identifying the prevalence of diabetes in a population, the MOHME emphasizes the use of self-reported use of anti-diabetes medication more than the self-reports of diabetes as a superior definition. The alternative definitions provided in this article also help us understand the sensitivity of the point estimates to the choice of definition. We considered patients with abnormally high FPG who responded “no” to the self-reported question as undiagnosed diabetes cases. The World Health Organization (WHO) and the American Diabetes Association (ADA) use a different threshold of FPG to define impaired fasting glucose (IFG).^10^ We, therefore, adopted a combination of the definitions to identify people having prediabetes when 1) 110 ≤ FPG < 126 mg/dL; 2) 100 ≤ FPG < 126 mg/dL; 3) 5.7 ≤ HbA1c < 6.5%, and, 4) 100 ≤ FPG < 126 mg/dL or 5.7 ≤ HbA1c < 6.5%. The WHO is the source of the first definition, and the other three definitions are those of the ADA.^10^ To measure blood glucose and lipid profile biomarkers in serum samples and HbA1c in whole blood, an autoanalyzer machine (Cobas C311 Hitachi High–Technologies Corporation, Tokyo-Japan) was used.

 To calculate the number of diabetes and prediabetes cases, we applied the sex- and age-specific estimated prevalence to the 2016 National Population and Housing Census enumerated by the Statistical Center of Iran.^11^ To calculate a wealth index for everyone in the original sample, we applied the Principal Component Analysis method to a set of standard questions on household asset ownership. We constructed the quintiles of the wealth index as a proxy for the ordered levels of socioeconomic status.^12^ We used STATA version 14 (College Station, TX: StataCorp LP) to produce the analytical file and survey package in R Version 3.4.0^13^ to generate the weighted prevalence estimates. A second analyst cross-checked the weighted prevalence estimates using the survey menu in STATA 14 to eliminate possible software dependency of the results. All reported P values were two-tailed. We considered *P* < 0.05 to be significant. The detailed information on the methodology of the 2016 STEPS, including the instructions for taking blood samples and calibration of the tests, can be obtained elsewhere.^6^

## Results

###  Prevalence of Diabetes and Prediabetes

 In 2016, 5 171 035 persons or 10.6% (95% CI: 10.0%–11.1%) of the Iranian population aged ≥ 25 years had diabetes according to the main definition: i.e. either high FPG or self-report of at least one anti-diabetes medication (1896 out of 18 947). However, the prevalence of diabetes in our sample changed according to different definitions and reached the highest 14.2% (95% CI: 13.6%–14.8%) according to either FPG or self-reported diagnosis of diabetes. Given the main definition of either high FPG or report of at least one anti-diabetes medication, the prevalence of diabetes was higher in females (11.2%; 95% CI: 10.5%–12.0%) than males (9.8%; 95% CI: 9.0%–10.6%). The univariable analysis showed that the prevalence of diabetes was 48% higher among urban dwellers than the rural residents, 15% higher among the wealthiest quintile than the people in the poorest quintile, and 71% higher in patients who had insurance than those without insurance. Also, those with less education uniformly had a higher prevalence of diabetes compared to more educated patients. Table 1 conveys detailed information on the demographic features of patients with diabetes in Iran.

**Table 1:** Prevalence (%) of Diabetes by Population Characteristics in 2016

**Characteristics**	**Only SR**	**SR or Medication**	**FPG (SR negative)**	**SR or FPG**	**FPG or Medication**
Gender					
Male	9.6 (8.8–10.4)	9.7 (8.9–10.5)	3.0 (2.5–3.4)	12.6 (11.7–13.4)	9.8 (9.0–10.6)
Female	13.0 (12.2–13.8)	13.1 (12.3–13.9)	2.6 (2.2–2.9)	15.6 (14.7–16.4)	11.2 (10.5–12.0)
Age (y)					
25–34	3.1 (2.5–3.7)	3.1 (2.5–3.7)	0.8 (0.5–1.2)	4.0 (3.2–4.7)	1.4 (1.0–1.7)
35–44	5.4 (4.7–6.1)	5.4 (4.7–6.2)	1.3 (0.9–1.6)	6.7 (5.9–7.5)	3.7 (3.1–4.4)
45–54	11.9 (10.6–13.2)	12.0 (10.7–13.3)	3.5 (2.8–4.1)	15.4 (14.0–16.8)	11.6 (10.3–12.9)
55–64	20.9 (19.2–22.7)	21.1 (19.4–22.9)	4.4 (3.6–5.3)	25.4 (23.5–27.2)	21.0 (19.3–22.8)
65–69	24.9 (21.6–28.3)	25.0 (21.7–28.4)	4.3 (2.7–5.9)	29.2 (25.7–32.8)	23.4 (20.1–26.6)
70 +	21.7 (19.2–24.2)	21.8 (19.3–24.3)	5.5 (4.3–6.7)	27.2 (24.6–29.8)	22.2 (19.7–24.7)
Residence					
Urban	12.5 (11.8–13.3)	12.6 (11.9–13.3)	3.0 (2.6–3.3)	15.5 (14.7–16.3)	11.7 (11.0–12.4)
Rural	9.0 (8.2–9.7)	9.0 (8.3–9.8)	2.2 (1.8–2.6)	11.2 (10.3–12.0)	7.9 (7.2–8.6)
Education					
None	17.4 (16.0–18.8)	17.6 (16.2–19.0)	4.6 (3.8–5.3)	22.0 (20.4–23.5)	17.5 (16.1–18.9)
1–6 years	13.0 (11.9–14.2)	13.1 (12.0–14.2)	2.7 (2.2–3.2)	15.7 (14.5–16.9)	12.0 (10.8–13.1)
7–12 years	9.2 (8.3–10.1)	9.2 (8.3–10.1)	2.2 (1.8–2.6)	11.4 (10.4–12.4)	7.9 (7.1–8.8)
> 12	7.9 (6.8–9.1)	8.1 (6.9–9.2)	2.1 (1.6–2.7)	10.1 (8.8–11.3)	7.2 (6.1–8.3)
Wealth index quintile					
Poorest	8.7 (7.7–9.7)	8.8 (7.8–9.7)	2.8 (2.2–3.4)	11.5 (10.4–12.6)	7.9 (6.9–8.9)
2	12.2 (11.0–13.5)	12.4 (11.2–13.7)	2.9 (2.3–3.5)	15.2 (13.8–16.5)	12.0 (10.7–13.2)
3	13.8 (12.4–15.3)	13.9 (12.4–15.4)	2.9 (2.3–3.5)	16.7 (15.2–18.3)	12.8 (11.4–14.2)
4	11.3 (10.1–12.5)	11.4 (10.2–12.6)	2.9 (2.3–3.6)	14.3 (12.9–15.6)	10.9 (9.7–12.2)
Richest	11.3 (10.0–12.7)	11.3 (10.0–12.7)	2.1 (1.6–2.7)	13.5 (12.0–14.9)	9.1 (7.9–10.4)
Health Insurance					
No	6.7 (4.8–8.5)	6.8 (5.0–8.6)	1.7 (0.8–2.6)	8.4 (6.3–10.4)	6.3 (4.6–8.1)
Yes	11.8 (11.2–12.4)	11.8 (11.3–12.4)	2.8 (2.5–3.1)	14.6 (13.9–15.2)	10.8 (10.3–11.4)
Total (25 + )^*^	11.5 (10.9–12.0)	11.5 (11.0–12.1)	2.7 (2.5–3.0)	14.2 (13.6–14.8)	10.6 (10.0–11.1)
Total (25 to 64)^**^	9.5 (9.0–10.1)	9.6 (9.0–10.1)	2.3 (2.1–2.6)	11.9 (11.3–12.5)	8.5 (8.0–9.1)
Total (25 to 70)^**^	10.7 (10.1–11.2)	10.7 (10.2–11.3)	2.5 (2.2–2.7)	13.1 (12.5–13.7)	9.6 (9.0–10.1)

FPG, fasting plasma glucose; SR, self-report. ^*^STEPS 2016 sample. ^**^Updated age group to compare current study with the previous STEPS for prevalence estimates.

 The national prevalence of prediabetes varied widely from 4.7% (95% CI: 4.3%-5.0%) based on 110 ≤ FPG < 126 mg/dL, to 31.2% (95% CI: 30.4%–32.0%) when we defined prediabetes as 100 ≤ FPG < 126 mg/dL or 5.7 ≤ HbA1c < 6.5%. With the latter definition, we estimated a total of 15 244 299 persons of the Iranian population aged ≥ 25 years having prediabetes (5885 out of 18 947). Using the ADA definitions, the prevalence of prediabetes by HbA1c was 5.4% greater than calculated using high FPG. Of note, the prevalence of prediabetes was 3.6 times higher with the minimum cutoff FPG set at 100 mg/dL (ADA definition) than when it was set at a minimum of 110 mg/dL (WHO definition). Regardless of the definition, prediabetes was more prevalent among males than females. Likewise, prediabetes showed an increasing prevalence in older age groups. Table 2 provides further details.

**Table 2:** Prevalence (%) of Prediabetes by Population Characteristics in 2016

**Characteristics**	**FPG (WHO)**	**FPG (ADA)**	**HbA1c (ADA)**	**FPG or HbA1c (ADA)**
	**110≤FPG<126 mg/dL** **(Excluding DM SR+or Insulin, Drug Takers)**	**100≤FPG<126 mg/dL** **(Excluding DM SR** **+or Insulin, Drug Takers)**	**5.7≤HbA1c<6.5%** **(Excluding DM SR+or Insulin, Drug Takers)**	**100≤FPG<126 mg/dL or 5.7≤HbA1c<6.5%** **(Excluding DM SR+or Insulin, Drug Takers)**
Gender				
Male	4.9 (4.3–5.4)	18.2 (17.2–19.2)	22.8 (21.8–23.9)	33.4 (32.3–34.6)
Female	4.5 (4.0–5.0)	15.4 (14.6–16.3)	21.4 (20.5–22.4)	29.3 (28.3–30.4)
Age (y)				
25–34	1.5 (1.1–1.9)	10.0 (8.9–11.0)	7.8 (6.8–8.7)	15.9 (14.6–17.2)
35–44	3.7 (3.0–4.4)	15.0 (13.8–16.2)	16.2 (14.9–17.5)	25.7 (24.2–27.2)
45–54	5.5 (4.6–6.4)	19.7 (18.1–21.2)	25.9 (24.3–27.5)	36.0 (34.2–37.8)
55–64	6.8 (5.8–7.9)	21.0 (19.2–22.8)	33.2 (31.3–35.2)	42.2 (40.1–44.3)
65–69	8.0 (6.1–9.9)	21.3 (18.1–24.5)	34.5 (30.8–38.2)	42.8 (39.0–46.6)
70 +	7.4 (6.0–8.7)	20.0 (17.9–22.1)	36.1 (33.4–38.7)	45.3 (42.5–48.0)
Residence				
Urban	4.8 (4.4–5.3)	17.1 (16.3–17.9)	21.5 (20.6–22.4)	30.6 (29.7–31.6)
Rural	4.3 (3.8–4.8)	15.7 (14.7–16.7)	23.3 (22.2–24.5)	32.4 (31.2–33.7)
Education				
None	6.6 (5.7–7.5)	18.4 (17.0–19.8)	33.5 (31.7–35.3)	41.6 (39.8–43.4)
1 – 6 years	5.1 (4.5–5.8)	18.6 (17.3–19.9)	24.1 (22.8–25.4)	33.7 (32.2–35.2)
7 – 12 years	4.0 (3.4–4.6)	15.9 (14.8–17.0)	17.9 (16.8–19.0)	27.4 (26.1–28.7)
> 12	3.4 (2.7–4.1)	13.4 (12.0–14.8)	16.4 (14.8–18.0)	24.9 (23.1–26.7)
Wealth index quintile				
Poorest	3.8 (3.2–4.4)	14.0 (12.8–15.3)	24.7 (23.2–26.3)	32.8 (31.1–34.4)
2	5.3 (4.5–6.1)	17.4 (16.0–18.8)	25.4 (23.7–27.0)	33.8 (32.1–35.6)
3	4.4 (3.7–5.1)	16.3 (14.9–17.6)	20.6 (19.1–22.1)	29.3 (27.6–31.0)
4	5.0 (4.1–5.8)	17.6 (16.1–19.1)	19.5 (18.0–21.0)	29.8 (28.1–31.6)
Richest	4.7 (3.8–5.5)	17.7 (16.1–19.3)	20.2 (18.5–21.8)	30.1 (28.2–32.0)
Health insurance				
No	4.8 (3.0–6.6)	17.7 (14.7–20.8)	19.1 (16.1–22.0)	29.1 (25.7–32.5)
Yes	4.7 (4.3–5.0)	16.6 (15.9–17.2)	22.3 (21.5–23.0)	31.3 (30.5–32.1)
Total (25 + )^*^	4.7 (4.3–5.0)	16.7 (16.0–17.3)	22.1 (21.4–22.8)	31.2 (30.4–32.0)
Total (25 to 64)^**^	4.2 (3.8–4.6)	16.0 (15.3–16.7)	19.8 (19.1–20.5)	29.0 (28.1–29.8)
Total (25 to 70)^**^	4.4 (4.0–4.8)	16.3 (15.7–17.0)	20.8 (20.1–21.5)	29.9 (29.1–30.7)

FPG, fasting plasma glucose; WHO, World Health Organization; ADA, American Diabetes Association; DM, diabetes mellitus; SR, self-report. ^*^STEPS 2016 sample. ^**^Updated age group to compare current study with the previous STEPS for prevalence estimates.

## Diabetes Control Achievements

 The median HbA1c for the age groups 25–44 years, 45–64 years, and 65 years and beyond were calculated as 5.3 (interquartile range [IQR]: 0.4), 5.6 (IQR: 0.7), and 5.8 (IQR: 0.8), respectively. Overall, 52.1% (95% CI: 49.4%–54.7%) of patients with self-reported diabetes were under strict glycemic control (HbA1c < 7%). Poor diabetes control (HbA1c > 9%) was found in 18.4% (95% CI: 16.3%-20.6%) of the patients who were aware of their diabetes. Strict diabetes control was achieved in 77.3% (95% CI: 72.6%–82.0%) of patients in the age group 25 to 44 years, while this achievement was shown in 48.1% (95% CI: 43.1%–53.1%) of patients aged at least 65 years. Overweight (25 ≤ BMI < 30) and obesity (BMI ≥ 30) were observed in 41.2% (95% CI: 38.6%–43.8%) and 36.1% (95% CI: 33.6%–38.5%) of patients with diabetes, respectively. Obesity was uniformly higher among women than men across different age groups. For instance, 39.5% (95% CI: 33.3%–45.8%) of women with diabetes aged at least 65 years were obese, while 18.5% (95% CI: 12.6%–24.3%) of men with diabetes in the same age group were shown to be obese. High blood pressure was under control (less than 130/80 mm Hg) in 34.0% (95% CI: 31.4%–36.5%) of the patients. Low LDL (low-density lipoprotein) cholesterol, indicated by LDL < 100 mg/dL, was measured in 58.1% (95% CI: 55.4%–60.7%) of patients with diabetes. Out of all patients in the sample, 6.6% (95% CI: 5.3%–8.0%) reported current daily cigarette smoking. Among all patients with diabetes, men aged between 45 and 64 years reported cigarette smoking more than 18 times that in women of the same age range. Low physical activity (less than 600 MET-minute per week) was observed in 60.4% (95% CI: 57.7%–63.0%) of the sample; women of all age groups showed uniformly lower physical activity than men. Self-reported adequate fruit and vegetable consumption, measured as greater than two servings of fruit and three servings of vegetable intake per day, was reported by 11.4% (95% CI: 9.6%–13.2%) of the patients. Of all the patients with diabetes, 73.6% (95% CI: 71.0%–76.2%) stated the current use of either oral anti-diabetes medication or insulin. Current insulin use, however, was reported in 15.7% (95% CI: 13.4%–18.0%) and daily testing of blood sugar at home in 13.0% (95% CI: 9.4%–16.5%) of the patients. Table 3 summarizes the results of diabetes control achievement in Iran.

**Table 3:** Prevalence (%) of Diabetes Control Achievement Indicators in Iran in 2016

**Indicators**	**Male**			**Female**			**Total**			**Grand Total**
	**25-44**	**45-64**	**≥65**	**25-44**	**45-64**	**≥65**	**25-44**	**45-64**	**≥65**	**All**
HbA1c										
> 9	13.1 (5.8–20.5)	20.3 (15.9–24.7)	25.0 (16.5–33.6)	8.6 (4.9–12.3)	20.1 (16.4–23.8)	16.7 (11.6–21.8)	10.1 (6.6–13.6)	20.2 (17.3–23.0)	20.1 (15.4–24.8)	18.4 (16.3–20.6)
< 8	82.5 (74.6–90.3)	64.3 (58.8–69.8)	62.3 (53.4–71.2)	86.4 (81.7–91.0)	62.3 (57.8–66.9)	67.8 (61.5–74.0)	85.1 (81.0–89.1)	63.1 (59.6–66.6)	65.5 (60.3–70.8)	67.6 (65.1–70.2)
< 7	72.6 (63.9–81.3)	44.2 (38.6–49.8)	45.1 (37.1–53.2)	79.7 (74.2–85.3)	47.2 (42.4–51.9)	50.1 (43.9–56.4)	77.3 (72.6–82.0)	46.0 (42.4–49.7)	48.1 (43.1–53.1)	52.1 (49.4–54.7)
BMI										
Underweight (BMI < 18.5)	2.7 (0.0–5.4)	0.8 (0.0–2.0)	1.9 (0.3–3.5)	1.8 (0.2–3.5)	0.3 (0.0–0.7)	0.6 (0.0–1.2)	2.1 (0.7–3.6)	0.5 (0.0–1.0)	1.1 (0.4–1.9)	1.0 (0.5–1.4)
Normal weight (18.5 ≤ BMI < 25)	31.1 (22.2–40.0)	23.3 (18.3–28.4)	32.6 (24.1–41.1)	25.2 (18.0–32.5)	14.3 (10.5–18.2)	21.5 (16.7–26.3)	27.3 (21.7–32.9)	17.7 (14.7–20.8)	26.1 (21.5–30.8)	21.8 (19.4–24.1)
Overweight (25 ≤ BMI < 30)	39.9 (29.8–50.0)	49.4 (43.6–55.2)	47.0 (38.7–55.3)	33.5 (27.0–39.9)	38.5 (33.9–43.0)	38.4 (32.1–44.7)	35.7 (30.1–41.2)	42.6 (39.0–46.2)	42.0 (36.9–47.0)	41.2 (38.6–43.8)
Obesity (30 ≤ BMI)	26.4 (17.9–34.9)	26.5 (21.8–31.3)	18.5 (12.6–24.3)	39.4 (32.5–46.4)	46.9 (42.2–51.5)	39.5 (33.3–45.8)	34.9 (29.5–40.4)	39.2 (35.7–42.7)	30.8 (26.2–35.3)	36.1 (33.6–38.5)
BP < 130/80	48.9 (38.9–58.9)	32.8 (27.4–38.1)	31.0 (22.4–39.6)	52.1 (45.0–59.2)	30.8 (26.3–35.3)	26.4 (20.4–32.4)	51.0 (45.2–56.8)	31.6 (28.1–35.0)	28.3 (23.3–33.3)	34.0 (31.4–36.5)
LDL < 100	69.0 (59.7–78.3)	59.4 (53.5–65.4)	64.9 (57.1–72.6)	56.4 (49.2–63.6)	53.1 (48.4–57.8)	59.1 (53.1–65.1)	60.6 (54.7–66.4)	55.5 (51.8–59.2)	61.4 (56.7–66.2)	58.1 (55.4–60.7)
LDL < 70 when CVD hx is +	1.9 (0.0–5.0)	17.6 (13.8–21.4)	27.0 (19.4–34.6)	4.2 (1.3–7.1)	14.3 (11.4–17.2)	21.1 (16.2–26.1)	3.4 (1.2–5.6)	15.6 (13.3–17.9)	23.5 (19.2–27.8)	15.8 (13.9–17.6)
Current daily smoking	14.4 (8.3–20.6)	17.8 (13.7–21.9)	11.4 (4.9–17.9)	1.2 (0.0–2.5)	1.0 (0.1–1.9)	2.0 (0.3–3.7)	5.6 (3.3–7.9)	7.4 (5.7–9.1)	5.9 (2.9–8.8)	6.6 (5.3–8.0)
Daily home-based testing (at least one time)	10.8 (0.0–23.2)	13.1 (5.8–20.5)	14.1 (5.3–22.8)	16.0 (3.5–28.5)	11.0 (6.4–15.6)	14.8 (2.4–27.1)	14.2 (4.9–23.4)	11.9 (7.8–16.0)	14.4 (6.9–22.0)	13.0 (9.4–16.5)
Low physical activity ( < 600 METs)	51.0 (40.0–61.9)	46.3 (40.5–52.2)	54.2 (45.5–62.9)	63.0 (55.7–70.4)	63.0 (58.4–67.5)	75.6 (69.7–81.4)	59.4 (53.3–65.4)	56.9 (53.2–60.6)	67.1 (62.1–72.2)	60.4 (57.7–63.0)
More than 2 servings of fruit and 3 servings of vegetable intake per day	18.0 (10.0–26.1)	14.8 (11.2–18.4)	14.2 (7.6–20.9)	8.8 (4.9–12.6)	9.9 (6.7–13.1)	7.8 (3.1–12.6)	11.9 (8.1–15.7)	11.8 (9.4–14.2)	10.5 (6.6–14.4)	11.4 (9.6–13.2)
Current insulin use	11.9 (3.1–20.8)	13.8 (9.4–18.2)	14.3 (9.1–19.4)	8.8 (4.2–13.4)	18.4 (14.0–22.8)	18.2 (11.9–24.5)	10.0 (5.5–14.5)	16.6 (13.4–19.8)	16.6 (12.2–20.9)	15.7 (13.4–18.0)
Current medication use	38.6 (26.1–51.0)	70.3 (64.0–76.7)	78.5 (71.8–85.1)	37.0 (28.5–45.6)	73.4 (68.7–78.1)	76.3 (69.7–83.0)	37.6 (30.5–44.7)	72.2 (68.4–76.0)	77.2 (72.4–82.0)	68.7 (65.9–71.6)
Current insulin or medication use	44.3 (31.8–56.8)	77.1 (71.1–83.1)	80.9 (74.4–87.3)	41.1 (32.5–49.7)	76.9 (72.4–81.4)	83.1 (78.2–87.9)	42.4 (35.2–49.6)	77.0 (73.3–80.6)	82.2 (78.3–86.1)	73.6 (71.0–76.2)

## Discussion

 In 2016, 10.6% of the Iranian population aged 25 years and more were estimated to have diabetes according to high FPG or report of anti-diabetes medication. Replacing the self-report of the anti-diabetes medications with the self-report dramatically increased the estimated cases with diabetes. It is unknown which definition of diabetes accurately reflects the true case prevalence. Consistent with the previous STEPS studies,^14^ the prevalence of diabetes correlated with age in both sexes. The urban to rural dominance in the prevalence has been stable since the first STEPS study in 2005. Our comparisons with previous STEPS are not age-standardized, but given the short period of time, we assume that the age pattern difference might not be significant. In both China and India, the prevalence of diabetes is higher in males than females.^15,16^ The Middle East and North Africa (MENA) region of the WHO had the highest prevalence of diabetes in the world in 2019.^17^ A systematic review on the prevalence of type 2 diabetes mellitus in the Middle East countries revealed that it varied from 2.6% (95% CI: 2.5%–2.6%) to 21.9% (95% CI: 16.8%–17.5%) among this region’s countries regardless of diabetes definition.^18^ Iran, according to the latest report in the Diabetes Atlas, was the third country in the MENA region with the highest number of diabetes among the population aged 20-79 years following Pakistan and Egypt in 2019.^17^ Two critical publications from the developing world have contributed to our detailed understanding of the burden of diabetes and prediabetes. Anjana et al measured the prevalence of diabetes in a large population (15 provinces) of India between 2012 and 2015 and concluded that 7.3% of the adult population had diabetes.^16^ A similar study by Wang et al from China estimated the national prevalence of diabetes among the adult population at 10.9%.^15^ The prevalence of diabetes in the United States was measured at 9.4% among the adult population in 2015.^19^ The definition of diabetes in these three reports was based on the self-report of diabetes, the same as one of our definitions. In the United States, this prevalence is slightly higher in men than women presented in the latest national diabetes report.^19^ Like our findings in the current study, a higher prevalence of diabetes was reported in urban areas of China and India than their rural areas.^15,16^

 Among the population aged 25–64 years, the prevalence of undiagnosed diabetes has been consistently dropping across the three STEPS studies (3.6% in 2005 to 2.63% in 2011). The prevalence of undiagnosed diabetes among the 25–70-year old population was estimated at 2.71% in STEPS 2011.^14^ Using the same definition, we calculated the prevalence of undiagnosed diabetes at 2.5% in our data, denoting a further, although trivial, drop of approximately 8% since 2011. The undiagnosed diabetes rate in our study was very close to that of India (2.6%),^16^ notably higher than that of the United States (1.15%),^20^ and significantly less than that of China (6.9%)^15^ in a comparable population, time, and definition.

 The estimation of prediabetes was quite sensitive to the choice of the biological threshold and the method of measurement. When the threshold of FPG was set at 110 mg/dL according to the WHO’s definition, our estimate of the prevalence was 4.7%. With the ADA’s definition (FPG threshold of 100 mg/dL), the prevalence increased by 3.6 times. There is controversy among scientists regarding the right threshold for defining prediabetes cases.^9,21^ Therefore, a trend toward reporting both thresholds is emerging in the published literature. Using HbA1c thresholds from the ADA, the prevalence of prediabetes was higher than that based on the ADA’s FPG thresholds (22.1% vs. 16.7%). Also, a significant number of cases defined as having prediabetes by HbA1c measure had normal FPG and vice versa. While both methods of measurement are appropriate to identify prediabetes patients, the ADA has some preference for using HbA1c over FPG.^22^ Compared to 2011, the prevalence of prediabetes among patients aged 25–70 years measured as 100 ≤ FPG < 126 mg/dL increased from 14.6% to 16.3% in 2016. The prevalence was higher in males than females and in urban residents than rural residents in both years. In our study, the population prevalence of prediabetes among the adult population using the WHO criteria was lower in Iran (4.7%) than India, which was reported as 10.3% in 2012-2013.^16^

 Our comparable prediabetes estimate of 16.7% is much lower than that reported by Wang et al. They reported the population prevalence of prediabetes as 35.7% in the Chinese adult population with at least 18 years of age in 2013 using the ADA definition.^15^ Likewise, the 2016 prevalence of prediabetes among adults aged at least 25 years was remarkably lower than that reported by the Centers for Disease Control and Prevention as 33.9% for the adult population of the United States aged at least 18 years in 2015.^19^

 Data on the quality of diabetes control are rarely published in developing countries. The STEPS 2016 study had a rich treasury of variables for analysis of the system’s achievements of diabetes control. We compare some of the results to those of the United States because these indicators have been periodically published in the peer-reviewed literature from the NHANES biannual survey data.^9^ Strict diabetes control (HbA1c < 7%) was observed in 52.1% of our weighted sample (age at least 25 years), while this value was calculated as 54.4% for the US adults (age at least 20 years) in NHANES 2013-2014. In our analysis, 18.4% of the weighted sample showed poor diabetes control (HbA1c > 9%). The corresponding value from the same US database was 15.0%.^9^ While in the NHANES data, strict diabetes control was achieved somewhat higher for the elderly than for younger age groups, this age gradient was reversed in our analysis as 77.3% of the sample aged between 25 and 44 years achieved strict diabetes control in Iran. This issue is particularly interesting as strict diabetes control is loosened in the official guidelines for controlling diabetes in the United States and worldwide, mainly due to the high risk of hypoglycemia as a result of excessive medications.^23,24^ Our estimates showed that 34.0% of patients with diabetes had blood pressure less than 130/80 mm Hg. Analysis of NHANES 2013-2014 revealed that in the United States, 48.8% of these patients achieved the same level of blood pressure control.^9^ LDL < 100 mg/dL, one of the targets for diabetes control, was achieved in 58.1% of the Iranian population vs. 53.3% in the US population in 2014.^9^ One possible reason behind this achievement is the high use of statins among the Iranian population.^25^ While self-reported use of oral anti-diabetes medications was comparable between the two databases (68.7% in STEPS 2016 and 68.4% in 2013–2014 NHANES), Iranian patients with diabetes reported the use of insulin significantly less than their American counterparts (15.7% vs. 28.6%).^9^ Anti-diabetes medications, including insulin, are widely available for use in the country. Adherence to oral anti-diabetes and insulin in Iran has been shown to be comparable to that in Western countries.^26,27^

 Overall, diabetes control achievement measures have not attained the level of those in the United States. The country has built the infrastructure to improve diabetes care, beginning almost two decades ago. The country has gone through several reforms to improve diabetes care. The National Program for Prevention and Control of Diabetes (NPPCD) was developed between the years 1999 and 2002. The first phase of the NPPCD was fully operationalized in rural areas in 2004 thanks to the strong primary care network with more than 40 000 community health workers, well-known as *Behvarz*. The main objectives of the first phase of the plan were active screening of diabetes in at-risk populations over 30 years and pregnant women and promoting the standard of diabetes care. The second phase of the NPPCD began in 2010 and planned to screen the population in six major metropolitan cities by passive and opportunistic screening of high-risk individuals. Enrolled patients went through annual assessments of micro- and macro-vascular complications of diabetes.^8,28,29^ Farzadfar et al showed that diabetes control in rural areas improved more than urban areas.^30^ Invaluable details about NPPCD in Iran can be obtained elsewhere.^31^ In 2015, the Non-communicable Diseases Committee (INCDC) developed the Iranian National Action Plan for the Prevention and Control of Non-Communicable Diseases. This multi-sectoral plan is aimed to control diabetes on both national and sub-national levels^28^ and is an extension of the previous policies for diabetes control. National Service Framework for Diabetes is a practical roadmap to expand the coverage of services for patients for diabetes and guarantees insurance coverage for all necessary medications and diagnostics for patients with diabetes. This new framework is yet to be fully implemented.

 While the prevalence of diabetes and prediabetes in Iran has been continuously increasing in both genders since our first documented national estimates in 2005, national plans for controlling diabetes and its outcomes have been taking effect around the same time. Given the high illness and cost burden of diabetes and prediabetes, these programs should continue to be considered among the highest priority policies. Likewise, continued research on the coverage and effectiveness of standard diabetes control programs is a national priority.
